# Cervical Esophago-Gastric Tubes for Patients with Malignant Ascites

**DOI:** 10.1007/s11605-016-3211-2

**Published:** 2016-07-29

**Authors:** Diana H. Liang, Min P. Kim, Edward Y. Chan, Puja Gaur

**Affiliations:** 1 Division of Thoracic Surgery, Department of Surgery, Houston Methodist Hospital, 6550 Fannin Street, Suite 1661, Houston, TX 77030 USA; 2 Weill Cornell Medicine, Houston Methodist Hospital, Houston, TX USA

**Keywords:** Malignant small bowel obstruction, Palliative decompression, Quality of life

## Abstract

Patients with chronic small bowel obstruction and malignant ascites from diffuse peritoneal carcinomatosis have limited options for gastrointestinal decompression as part of end-of-life palliation. Insertion of a percutaneous gastrostomy tube is relatively contraindicated in patients with ascites. Alternatively, nasogastric tube placement often leads to significant discomfort to patients and necessitates hospitalization during their last days of life. Here, we demonstrate how placing a percutaneous cervical esophago-gastric tube can allow adequate gastrointestinal decompression for terminal patients with malignant small bowel obstruction. This palliative measure allows them to remain in the comfort of their own homes after the procedure.

**Electronic supplementary material:**

The online version of this article (doi:10.1007/s11605-016-3211-2) contains supplementary material, which is available to authorized users.

## Introduction

Malignant small bowel obstruction (MSBO) is a common diagnosis in patients with advanced stage malignancies.^1^ Bypass surgery is the standard surgical palliative treatment for these patients; however, palliative surgery in these terminal patients is often not well-tolerated.^1^,^2^ In these patients, palliative decompression of MSBO to avoid intractable vomiting is necessary. While these patients are admitted, nasogastric tubes (NGTs) are often placed for immediate decompression of the stomach. However, NGTs are poorly tolerated as they cause significant discomfort to patients and may even cause sinusitis and pressure ulcers of the nasal septum.^3^ In addition, NGTs commit most patients to spend their last few days of life in the hospital since discharging them home risks dislodgement of the tube. Therefore, percutaneous endoscopic gastrostomy (PEG) tube has been advocated for gastrointestinal decompression to allow end-of-life care and palliation at home.^2^ However, often times in patients with MSBO and diffuse peritoneal carcinomatosis, the presence of ascites makes PEG placement technically difficult, as ascites fluid limits the degree of transillumination through the stomach, impairs maturation of the fibrous tract along the PEG tube,^4^ and sets the patient up for intraperitoneal contamination as well as persistent ascites leakage from the PEG tube site.^4^ Although large-volume paracentesis prior to PEG tube insertion and at intervals thereafter may reduce the likelihood of peristomal ascitic fluid leakage,^2^ ascites have been viewed as a relative contraindication to PEG placement.^5^

In an effort to allow end-of-life care at home for patients with ascites associated with MSBO, over the last 2 years we have successfully placed three percutaneous palliative cervical esophago-gastric tubes in terminal patients to decompress their gastrointestinal tract.

## Technique

**Fig. 1:**
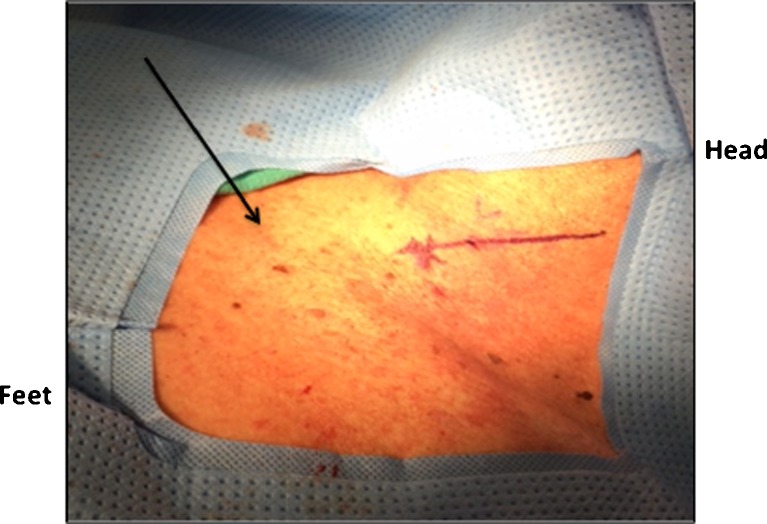
Landmark of needle puncture site, anterior to the sternocleidomastoid muscle in left cervical region about two fingerbreadths above the sternoclavicular joint (blackarrow)

**Fig. 2:**
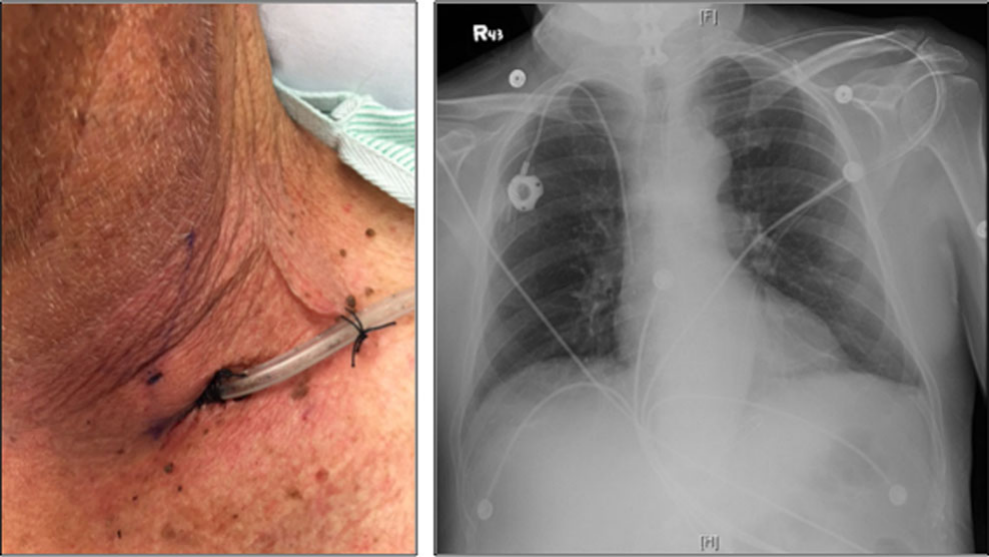
Final image of percutaneous cervical esophago-gastric tube placement along with radiographic image

The procedure is performed under general anesthesia in a supine position in the operating room. The patients’ neck is turned to the right and their left neck is prepped and draped in a routine sterile fashion. With or without ultrasound guidance (operator preference), a 21-gauge introducer needle from a Micropuncture® Introducer Set (Cook Medical, Bloomington, IN) is inserted following a track anterior to the sternocleidomastoid muscle (Fig. 1). During the insertion, suction is applied onto the needle to prevent any injury to major vessels in the neck. Intubation of needle into the esophagus is confirmed with simultaneous endoscopy. With the needle in the esophagus, the guidewire from the Micropuncture® Introducer Set is then threaded down under endoscopic guidance. The introducer needle is then removed and the tract is dilated with a 5 Fr dilator from the Introducer set. The wire is removed, and the Amplatz Super Stiff^TM^ wire (Boston Scientific, Marlborough, MA) is passed distally into the esophageal lumen. The dilator is withdrawn, and a 22 Fr dilator with a peel-away sheath (Kimberly-Clark, Roswell, GA) is then used to dilate the tract after enlarging the neck incision. The Amplatz Super Stiff^TM^ wire and dilator are removed, leaving the peel-away sheath in place. An 18 Fr NGT is then threaded through the sheath, which is then separated leaving the NGT in place. The enteric tube is guided into the proximal stomach under endoscopic visualization, and is anchored to the neck at approximately 40 cm using 2–0 silk sutures (Fig. 2). Two stay sutures are placed along the patient’s left side of the neck to anchor the tube without tension.

## Discussion

Despite the common occurrence of MSBO in patients with advanced stage cancer, there has not been any significant improvement in its prevention or management.^1^ Because the majority of available treatments are futile at this stage of disease, improving quality of life for such terminal patients is of utmost importance. Currently utilized NG and PEG tubes as a palliative measure are less than optimal methods for these patients. Therefore, we have successfully employed a novel technique as described here in three patients with MSBO and malignant ascites from diffuse peritoneal carcinomatosis. One of our patients with metastatic rectal cancer developed a skin abscess around the cervical esophago-gastric tube on postoperative day 2 that resolved by postoperative day 7 with a counter-incision and drainage at the bedside and antibiotics. It was felt that the abscess occurred as the patient was on concurrent chemotherapy and thus immunosuppressed. In the other two patients (with metastatic gastric cancer and colon cancer), there were no procedure-related complications, and all of them did well. All of our patients were discharged home within 2 weeks after the procedure with no re-admission to the hospital. The patient’s fluid shifts and pain were managed at home under the care of a hospice team.

Based on our experience, we feel that surgeons can easily adopt this technique. Cervical esophago-gastric decompression is well-tolerated by patients and is easily managed by their families. Therefore, we advocate the use of cervical esophago-gastric decompression in terminal patients with MSBO for palliation, which can allow them to be home for hospice care.

## Electronic Supplementary Material

Below is the link to the electronic supplementary material.

Video 1 Step-by-step technique for placement of the cervical esophago-gastric tube. (MOV 15660 kb)
